# Scientists’ Reputations Are Based on Getting It Right, Not Being Right

**DOI:** 10.1371/journal.pbio.1002460

**Published:** 2016-05-12

**Authors:** Charles R. Ebersole, Jordan R. Axt, Brian A. Nosek

**Affiliations:** 1 University of Virginia, Psychology Department, Charlottesville, Virginia, United States of America; 2 Center for Open Science, Charlottesville, Virginia, United States of America

## Abstract

Replication is vital for increasing precision and accuracy of scientific claims. However, when replications “succeed” or “fail,” they could have reputational consequences for the claim’s originators. Surveys of United States adults (*N* = 4,786), undergraduates (*N* = 428), and researchers (*N* = 313) showed that reputational assessments of scientists were based more on how they pursue knowledge and respond to replication evidence, not whether the initial results were true. When comparing one scientist that produced boring but certain results with another that produced exciting but uncertain results, opinion favored the former despite researchers’ belief in more rewards for the latter. Considering idealized views of scientific practices offers an opportunity to address incentives to reward both innovation and verification.

## Abstract 2

How are scientists evaluated? Members of the general public, undergraduate students, and active researchers all agree that the research process, not the results, determines a scientist’s reputation.

## Introduction

With increasing attention paid to reproducibility in science [1,2], a natural worry for researchers is, “What happens if my finding does not replicate?” We might ask ourselves that question out of intellectual curiosity but also out of concern for our reputations. Indeed, one failure to replicate in a special issue of preregistered replications in *Social Psychology* [3] sparked “repligate” on social media, with accusations, name-calling, and the general sense that replication is fraught with social consequences well beyond the scientific implications of the research [4].

In a dispassionate scientific enterprise, whether replications “succeed” or “fail” only has consequences for the finding being studied and the methodology being used to study it. But doing science is not dispassionate. Researchers study topics they find interesting, develop theories they believe in, and publish findings that become part of their identities. In a sense, researchers treat their findings like possessions [5]. If we feel ownership of our findings, failures to replicate can threaten not just the finding but also our status as its discoverer and as a competent scientist. It is no wonder that researchers perceive failures to replicate as a threat to both the result in question and the reputation of its originator [6].

## Weighing Innovation against Reproducibility

Many would argue that scientists should be evaluated only for the things that they can control. Researchers control the question, hypothesis, design, implementation, analysis, and reporting. Researchers are not supposed to control the results. Of course, it is not that simple. Results are determined by reality, but scientists generate the ideas and insights that enable the discovery of those results. As such, scientific contributions may be evaluated by (1) the degree to which the results are exciting, innovative, and pushing into new areas for knowledge accumulation and (2) the degree to which the results are certain, reproducible, and true.

Exciting, innovative results are better than boring, incremental results, and certain, reproducible results are better than uncertain, irreproducible results. However, a result that is both innovative and certain is a rarely achieved ideal. If you had to choose between them, which would you pick? Considering this tradeoff may reveal the scientific culture, identify factors that influence research planning, and foster discussion about how best to align scientific incentives with scientific values.

The present culture in science provides strong incentives for innovation and relatively weak incentives for certainty and reproducibility [7,2]. This suggests that the recipe for reputation and career success is to prioritize innovative ideas over reproducible evidence. However, the weak incentives for reproducibility have raised concerns about the credibility of the published record [8,1] and are at odds with the perceived core values of science that scientists themselves endorse [9].

**Fig 1:**
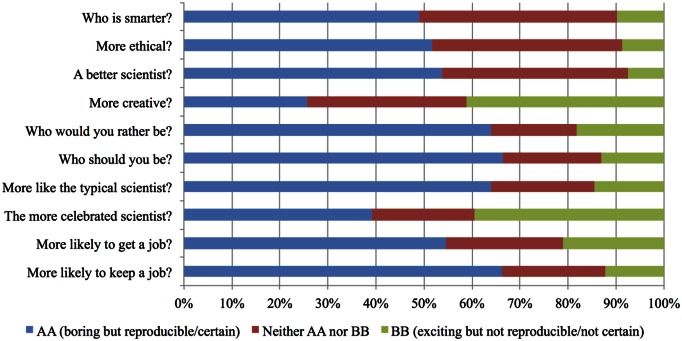
Respondents imagined two scientists AA and BB who demonstrate different characteristics in the results that they produce from their research. AA produces boring but certain results; BB produces exciting but uncertain results (Total *N* = 4,786). Fig 1 data can be accessed at https://osf.io/wqxjn/. Note: Figure aggregates two data collections. For one (*N* = 1,321), “certain” and “uncertain” were replaced with “very reproducible” and “not very reproducible,” and a definition was provided: “Reproducible means that the results recur when the study is conducted again.” Results were very similar between phrasings.

What are the idealized expectations of scientists in the public sphere? To examine values about science and scientists, we presented members of the general population with a stark choice between two extremes. Scientist AA produces “boring but certain” results; Scientist BB produces “exciting but uncertain” results. We did not define the scientists’ discipline or expertise. We collected two large, heterogeneous samples of US adults via an online sampling firm (*N* = 1,321 and *N* = 3,465). For the larger sample, we used the terms “certain” and “uncertain”; for the smaller sample, we replaced those terms with “very reproducible” and “not very reproducible.” Results were very similar across terminologies and samples (see S1 Text), so the aggregate results are presented in Fig 1.

Respondents evaluated the scientist who produces boring but certain (or reproducible) results more favorably on almost every dimension compared to the scientist who produces exciting but uncertain (or not reproducible) results. The reproducible researcher was perceived as smarter, more ethical, a better scientist, more typical, and more likely to get and keep a job. Respondents also reported that they would want to be and should be more like the reproducible researcher. At the same time, more respondents reported that the exciting researcher is more creative and would be more celebrated than the reproducible researcher (See S1 Text for summary statistics for all measures).

## Replication and Reputation

When confronted with a stark tradeoff, respondents prioritized certainty and reproducibility over exciting results. However, in reality, scientists do not aim for boring results in order to achieve reproducibility. The degree to which ideas are exciting is usually known first; certainty comes later. As a consequence, this idealization is not as much about selecting effects to study as it could be about planning a research program. After obtaining an exciting result, should we publish it and move on to chase the next exciting finding, or should we work to achieve greater certainty via replication and other strategies? And considering the reputational stakes, how should we respond when others attempt to replicate our findings to increase certainty independently?

**Fig 2:**
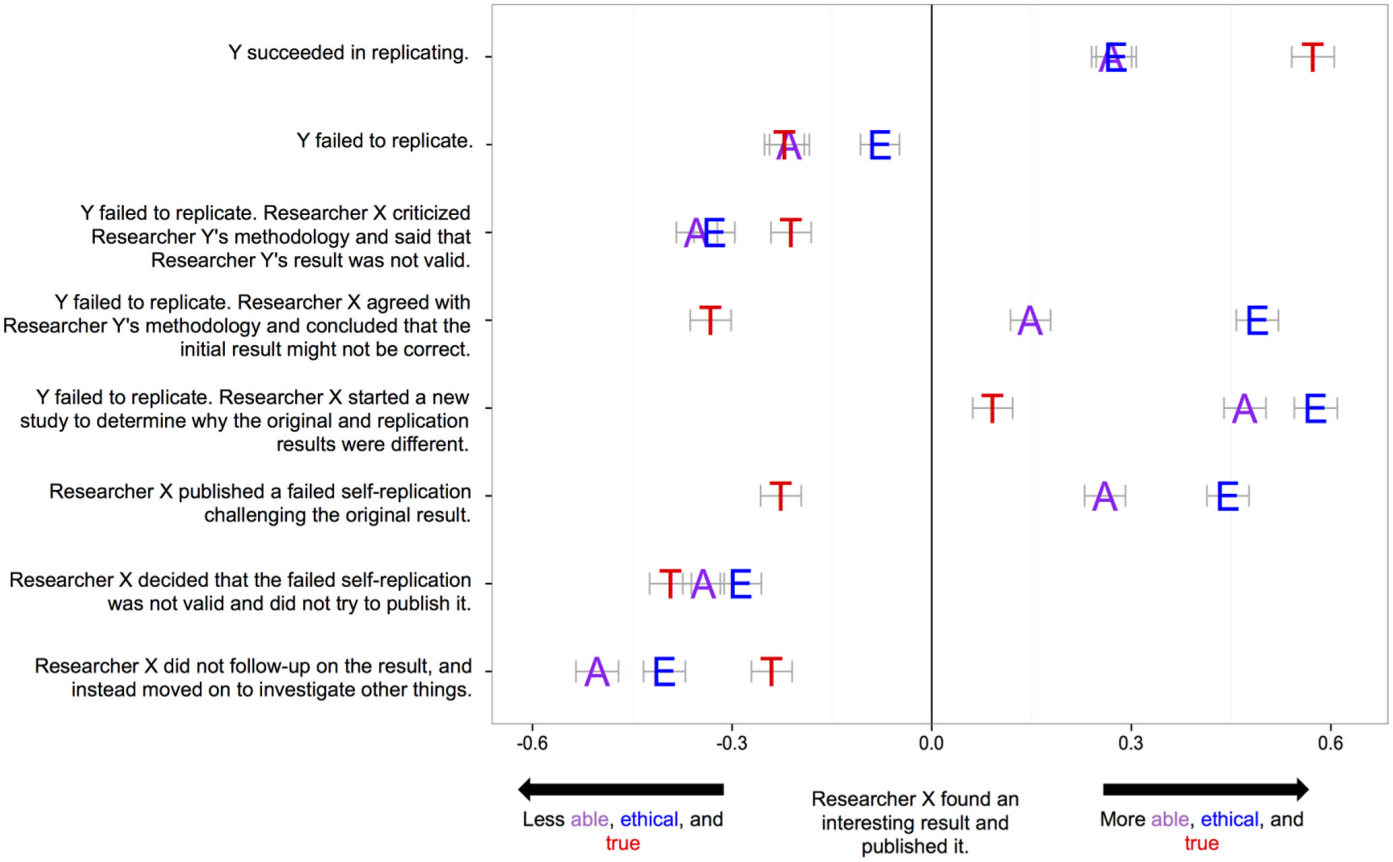
Effect of replication on perceived ability (purple) and ethics (blue) of Researcher X and truth (red) of the original result (*N* = 4,786). Error bars indicate 95% confidence intervals of the effect size (Cohen’s d units, maximum CI width = .065) of difference between scenario judgments versus the baseline “Researcher X found and published an effect.” Fig 2 data can be accessed at https://osf.io/wdfp8/.

To investigate reactions to these issues, the same respondents completed another survey in which they read a general descriptive paragraph of the scientific and publishing process before learning that “Researcher X did a study and found an interesting result and published it.” With this minimal information, participants evaluated Researcher X on ability (*M* = −0.03, *SD* = 2.05, Range −6 [“one of the worst researchers ever”] to 0 [“about the same as the average researcher”] to +6 [“one of the best researchers ever”]) and ethics (*M* = −0.26, *SD* = 2.07, Range −6 [“one of the least ethical researchers ever”] to 0 [“about the same as the average researcher”] to +6 [“one of the most ethical researchers ever”]). Participants also rated the likelihood of the result being true (*M* = −0.05, *SD* = 1.68, Range −5 [“definitely incorrect”] to 0 [“about equally likely to be correct or incorrect”] to +5 [“definitely correct”]). Then, respondents evaluated Researcher X and the finding on those same three dimensions after various outcomes (See S1 Text for summary statistics for all measures). Fig 2 presents the extent to which perceived ability, ethics, and truth of the result increased or decreased for each of eight events that followed the initial discovery and publication. For example, in the first row, after Researcher X found an interesting result and published it, Researcher Y followed up on the result and successfully replicated it. Perception of Researcher X’s ability (*d* = .27), ethics (*d* = .28), and perceived truth of the result (*d* = .57) increased compared to baseline.

In rows 2 through 5, Researcher Y failed to replicate Researcher X’s result, but evaluations of ability and ethics varied substantially from perceptions of truth depending on Researcher X’s reaction. Perceived ability and ethics dropped slightly to moderately with Y’s failure to replicate, particularly if X criticized the replication. Notably, perceptions of X’s ability and ethics increased in the face of a failed replication if X agreed that the original result might not be correct or if X did a follow-up study to determine why different results were observed. In fact, the increases in perceived ethics (and ability when conducting a follow-up) even exceeded the reputational benefits of an independent, successful replication (row 1). Moreover, when X responded to the failed replication by conducting a follow-up study, perceived truth of the original result even increased slightly from baseline without respondents knowing the outcomes of the follow-up research.

In rows 6 and 7, there was no Researcher Y. Researcher X failed to replicate the original result him- or herself. In both cases, perceived truth of the result declined, but evaluations of ability and ethics depended strongly on how Researcher X reacted. Publishing the failed self-replication increased perceived ability (*d* = .26) and ethics (*d* = .44), and dismissing it decreased perceived ability (*d* = −.34) and ethics (*d* = −.29).

Finally, in row 8, Researcher X published the original result and then decided to just move on to study other research questions instead of following up on the original result. This decision decreased perceived ability (*d* = −.50), ethics (*d* = −.40), and truth of the result (*d* = −.24). Publishing a result and moving on was evaluated as indicating the least ability and ethics, even compared to the scenarios in which the study failed to replicate.

Reputation—as indexed by perceived ability and ethics—did not mirror assessments of the truth of the original result. Researchers’ reputations were more closely tied to their process whether they were responding to others’ replications or pursuing their own. Reputations even increased if self-replication failure was reported or if other-replication failure was pursued with follow-up research. This opposes the notion that failure to replicate, in and of itself, is a threat to reputation. In the abstract, pursuing truth is valued more than being validated for having found it.

## Scientific Ideals among Researchers

These surveys illustrate trade-offs among scientific values that members of the general population place on scientific practices. Those more closely involved with research might assess those responses as overly idealistic and demur that science does not actually work that way. However, when given the same measures, undergraduate students (*N* = 428) and active psychology researchers (*N* = 313; 50.4% faculty, 14.1% postdocs, and 29% graduate students) reported similar opinions across both surveys (see S1 Text, Studies 2 and 4, and also [6]). For example, like the general population, researchers themselves evaluated a researcher’s ability and ethics strongest when he or she had a finding that failed to replicate and either agreed with it, followed-up to investigate why, or published his or her own failure to replicate. An important limitation of the results from undergraduates and researchers is that they are comparatively small samples and not representative of those two populations. Replication of these results with large, representative samples will be very useful for obtaining precise, generalizable estimates.

Researchers differed most from the general population in being forgiving of the researcher that did not follow-up on an initial published study, perhaps in recognition of the strong incentives pushing for innovation. A similar difference between researchers and the general population was observed in the second survey. More researchers rated a scientist who produces boring but certain findings to be a better and more ethical scientist than one who produces exciting but uncertain findings by a wide margin. However, unlike the general population, researchers assessed the exciting, uncertain scientist as more likely to get a job, keep a job, and be more celebrated by wide margins. Despite that, researchers were slightly more likely to say that they would rather be, and more than twice as likely to say that they should be, the boring, certain scientist. Undergraduates’ responses more closely mirrored those of the general population than scientists. These results suggest that scientists embrace the same ideals as the general population and undergraduate students but simultaneously perceive the cultural mechanisms of reward and advancement as favoring beauty and innovation over accuracy [7,9,10].

Despite the incentives against them, there have been notable illustrations of these reported ideals in practice. For example, LeBel and Campbell [11] reported two failures to replicate an effect reported by Vess [12]. When the journal editor reached out to Matthew Vess to write a rejoinder, he responded with the following:

Thank you for the opportunity to submit a rejoinder to LeBel and Campbell's commentary. I have, however, decided not to submit one. While I am certainly dismayed to see the failed attempts to reproduce a published study of mine, I am in agreement with the journal's decision to publish the replication studies in a commentary and believe that such decisions will facilitate the advancement of psychological science and the collaborative pursuit of accurate knowledge. LeBel and Campbell provide a fair and reasonable interpretation of what their findings mean for using this paradigm to study attachment and temperature associations, and I appreciated their willingness to consult me in the development of their replication efforts.

Vess captures the stakes both for himself and for the discipline, distinguishes them clearly, and promotes the transparent dissemination and debate of replication in service of accumulating knowledge. Informally, it is difficult to imagine this stance leading to his diminished reputation as a scientist. Further, the “repligate” furor focused on debate about a single article from the special issue of *Social Psychology* and became more about the tone with which replications are discussed rather than the replications themselves. Reactions to the 14 other articles, most of which included failures to replicate, followed a routine pattern of scientific debate (see all preregistrations, articles, commentaries, and rejoinders at https://osf.io/hxeza/wiki/replications/) and even inspired follow-up investigations by some original authors. It is easy to get caught up in the drama of contested replications and miss the more prevalent, constructive, even pallid academic debate about replication results in the service of knowledge building.

## The Reality of Replication and Reputation

Our surveys obtained evaluations of specific behaviors sterilized from the messiness of reality. A strength of this approach is isolating the impact of particular behaviors on evaluation of desirability, ethics, ability, and truth. A weakness of this approach is that reality is more complicated, and the impact of innovation and reproducibility on reputation is most certainly moderated by factors beyond what we studied. For example, our evidence suggested that criticizing replications has a reputational cost for original authors. Given our design, that conclusion is contingent on knowing no other information about the situation. Under what conditions might criticizing the replication not impact, or even enhance, the reputation of the original authors? It is easy to generate possible scenarios such as evidence that the replication team had a conflict-of-interest or ideological opposition to the original finding. It is also easy to generate possible scenarios in which reputation would worsen even further—such as the original authors accusing the replication team of a conflict-of-interest or ideological opposition, but with no independent evidence that this is the case.

The fact that human behavior is more complicated than what we studied is a trite truism. Behavioral research advances incrementally by unpacking a complex phenomenon to identify mechanisms, moderating influences, and boundary conditions for how it works. Some obvious extensions of the present evidence could investigate the impact of replication on the reputation of the replicators, the impact of replication on reputation when “camps” exist defending or opposing a particular finding, the impact of the quality of evidence in the original and replication studies, the impact of the preexisting reputation of the original authors and replicators, and how these reputational stakes and tradeoffs among scientific values might vary across scientific disciplines, particularly mature versus breakthrough areas of study.

## Conclusion

Well over 1 million articles are published each year [13], and only a small fraction represent true innovative breakthroughs. Over time, many new and exciting findings will be identified as false leads. Even so, aiming for the next breakthrough result is not a fool’s errand. Taking risks with what could be true seeds the marketplace of ideas with new directions. If we are not wrong frequently, we are failing to push on the frontiers of knowledge hard enough. At the same time, because true innovations are rare, valuing replication will foster efficient filtering of interesting findings and accelerate knowledge building. Otherwise, researchers may persist in a continuous generation of innovative results without complementary efforts to verify them. Moreover, as our survey data suggest, replication need not be fraught with concerns about reputation. In fact, more rapid identification of false leads allows one to focus resources on the ideas that are surviving the scrutiny. In a scientific culture valuing both innovation and verification, we will not stop aiming for the rewards of beautiful results, but we will be more confident that we earned them.

## Supporting Information

S1 Text This file contains all Supporting Information, including extended descriptions of the methods and results of the four data collections referenced in this paper. **Study 1**: Detailed description of sample, methods, and results from Study 1 (pp. 1–6). **Table A:** Responses to replication, descriptive statistics, Study 1. **Table B:** Attributions of researchers, Study 1. Number of participants selecting AA, BB, or neither. **Study 2**: Detailed description of sample, methods, and results from Study 2 (pp. 6–9). **Table C:** Responses to replication, descriptive statistics, Study 1. **Table D:** Attributions of researchers, Study 1. Number of participants selecting AA, BB, or neither. **Study 3**: Detailed description of sample, methods, and results from Study 3 (pp. 9–12). **Table E:** Responses to replication, descriptive statistics, Study 1. **Table F:** Attributions of researchers, Study 1. Number of participants selecting AA, BB, or neither. **Study 4**: Detailed description of sample, methods, and results from Study 4 (pp. 12–15). **Table G:** Responses to replication, descriptive statistics, Study 1. **Table H:** Attributions of researchers, Study 1. Number of participants selecting AA, BB, or neither.
(DOCX)
