# Supplementary material for: Scientists’ Reputations Are Based on Getting It Right, Not Being Right
Source: PLoS Biol. 2016 May 12;14(5):e1002460. doi: 10.1371/journal.pbio.1002460 (PMC4865149; doi:10.1371/journal.pbio.1002460)
Supplement: S1 Text — Study 1: Detailed description of sample, methods, and results from Study 1 (pp. 1–6). Table A: Responses to replication, descriptive statistics, Study 1. Table B: Attributions of researchers, Study 1. Number of participants selecting AA, BB, or neither. Study 2: Detailed description of sample, methods, and results from Study 2 (pp. 6–9). Table C: Responses to replication, descriptive statistics, Study 1. Table D: Attributions of researchers, Study 1. Number of participants selecting AA, BB, or neither. Study 3: Detailed description of sample, methods, and results from Study 3 (pp. 9–12). Table E: Responses to replication, descriptive statistics, Study 1. Table F: Attributions of researchers, Study 1. Number of participants selecting AA, BB, or neither. Study 4: Detailed description of sample, methods, and results from Study 4 (pp. 12–15). Table G: Responses to replication, descriptive statistics, Study 1. Table H: Attributions of researchers, Study 1. Number of participants selecting AA, BB, or neither. (DOCX) [file pbio.1002460.s001.docx]

**Study 1**

**Method**

**Consent Form**

Prior to participation, all participants saw the following consent form:

Informed Consent Agreement: Reputations of Scientists

Please read this consent agreement carefully before you decide to participate in the survey.

Purpose of the research survey: The purpose of this survey is to learn about how scientists judge other scientists.

What you will do in the survey: In this survey, you will read several scenarios and descriptions of scientists engaged in scientific discovery. You will be asked to judge these individuals based on their actions and characteristics. Then you will answer a few questions about yourself.

Time required: This survey will require about 10-15 minutes of your time.

Risks: There are no anticipated risks associated with participating in this survey.

Benefits: There are no direct benefits to you for participating in this research survey. At the end of the survey, we will provide an explanation of the survey and a link to a summary of results from U.S. adult samples. Also, if you would like to receive the results of this data collection, at the end of the study you can submit your email address (stored separately with no connection to your data) and the results will be sent to you.

Confidentiality: The information that you give in the survey will be handled confidentially. Your information will be assigned a code number. Your name will not be used in any report. After completion of the survey, versions of the data, with all identifying information removed, will be made available so that other researchers can evaluate it.

Voluntary participation: Your participation in the survey is completely voluntary.

Right to withdraw from the survey: You have the right to withdraw from the survey at any time without penalty.

How to withdraw from the survey: If you want to withdraw from the survey, simply close your browser window.

If you have questions about the survey, contact: Charlie Ebersole, Department of Psychology, University of Virginia, Charlottesville, VA 22904

Telephone: (937) 304-3175, Email: cre2am@virginia.edu

If you have questions about your rights in the survey, contact:

Tonya Moon, Ph.D., Chair, Institutional Review Board for the Social and Behavioral Sciences , One Morton Dr Suite 500 University of Virginia, P.O. Box 800392, Charlottesville, VA 22908-0392

Telephone: (434) 924-5999; Email: irbsbshelp@virginia.edu; Website: http://www.virginia.edu/vprgs/irb

Agreement: I agree to participate in the research survey described above.

By clicking the button below you are indicating that you have read the informed consent statements above and agree to participate.

**Participants**

Participants (*N* = 1,321) from the United States were recruited online through the sampling agency SoapBox Sample and paid in return for completing a 20 minute study. Overall, the sample was 67.8% female, the average age was 49 years (*SD* = 14.09), and 82% were White, 6.5% Black, 3.8% Asian, 4.4% Hispanic, and 3.3% Other. The sample is highly diverse, but the firm’s methodology does not produce a probability sample or a representative sample of U.S. residents.

**Materials**

Participants completed two sets of measures concerning the reputations of scientists.

**Responses to Replication**. The first set focused on perceptions of scientists responding to replications of their work. After reading a brief introduction, participants read nine possible scenarios that could occur during the scientific process.

The introduction provided context for the research process for participants: “On the following pages, you will read several scenarios and provide your impressions of people. Read each scenario carefully, but remember, we are interested in your impressions.

Scientists investigate questions for which the answer is not yet known. Scientists conduct studies, evaluate the results, and sometimes write up the results to report in a scientific journal to share what they have learned. Before publishing the report, editors at the journal send the report out to a few other scientists to evaluate the quality and value of the study and reported results. The editor considers the reviewers comments and accepts or rejects the report for publication. Once published, other scientists might read about the results and conduct additional research. For example, another scientist might try to repeat the experiment to see if the same result occurs, if it changes with differences in the procedure, or to explore whether the initial result occurred by chance. It is rare that any single study answers a scientific question. In most cases, many studies are done and evidence accumulates toward an answer.

Based on what you know about how science works, on the next pages, please rate your impression of researchers based on various behaviors or events that occur in science. You will receive 9 scenarios and answer the same 3 questions for each one.”

Participants then judged a hypothetical scientist (Researcher X) based on her/his research practices and responses to replications on three dimensions: the scientist’s ability as a researcher (-6 “*one of the worst researchers ever*”, 0 “*about the same as the average researcher*”, 6, “*one of the best researchers ever*”), how ethical the scientist is, (-6 “*one of the least ethical researchers ever*”, 0 “*about the same as the average researcher*”, 6, “*one of the most ethical researchers ever*”), and how likely it is that the original effect is true (-5 “*definitely incorrect*”, 0 “*about equally likely to be correct or incorrect*”, 5 “*definitely correct*”). All scale points were labeled. Participants read the following scenarios:

1. Researcher X did a study and found an interesting result and published it.
2. Researcher X did a study and found an interesting result and published it. Researcher Y tried to replicate the result and succeeded.
3. Researcher X did a study and found an interesting result and published it. Researcher Y tried to replicate the result and failed to reproduce it.
4. Researcher X did a study and found an interesting result and published it. Researcher Y tried to replicate the result and failed to reproduce it. Researcher X criticized Researcher Y's methodology and said that Researcher Y's result was not valid.
5. Researcher X did a study and found an interesting result and published it. Researcher Y tried to replicate the result and failed to reproduce it. Researcher X agreed with Researcher Y's methodology and concluded that the initial result might not be correct.
6. Researcher X did a study and found an interesting result and published it. Researcher Y tried to replicate the result and failed to reproduce it. Researcher X examined Researcher Y's methodology and started a new study to determine why the original and replication results were different.
7. Researcher X did a study and found an interesting result and published it. Later, Researcher X tried to replicate the result and failed to reproduce it. Researcher X published the replication challenging the original result.
8. Researcher X did a study and found an interesting result and published it. Later, Researcher X tried to replicate the result and failed to reproduce it. Researcher X decided that the failed replication was not valid and did not try to publish it.
9. Researcher X did a study and found an interesting result and published it. Later, Researcher X did not follow-up on the result, and instead moved on to investigate other things.

Participants saw scenario 1 first, then 2 and 3 in a random order, then 4-6 in a random order, and then 7-9 in a random order. Participants made the same three judgments of for each scenario.

**Attributes of Researchers**. The second set of measures concerned attributes of researchers based on the type of research they produce. Participants considered two researchers: AA, who produced boring results that are very reproducible, and BB, who produced exciting results that are not very reproducible.

Participants first received opening context: “Imagine two scientists AA and BB who demonstrate different characteristics in the results that they produce from their research. Reproducible means that the results recur when the study is conducted again.” Then, they selected AA, BB, or neither in response to 10 attributes presented in a fixed order:

1. Which scientist is smarter: AA who produces boring results that are very reproducible or BB who produces exciting results that are not very reproducible.
2. Which scientist is more ethical: AA who produces boring results that are very reproducible or BB who produces exciting results that are not very reproducible.
3. Which scientist is a better scientist: AA who produces boring results that are very reproducible or BB who produces exciting results that are not very reproducible.
4. Which scientist is more creative: AA who produces boring results that are very reproducible or BB who produces exciting results that are not very reproducible.
5. Which scientist would you rather be: AA who produces boring results that are very reproducible or BB who produces exciting results that are not very reproducible.
6. Which scientist should you be: AA who produces boring results that are very reproducible or BB who produces exciting results that are not very reproducible.
7. Which scientist is more like the most celebrated scientists: AA who produces boring results that are very reproducible or BB who produces exciting results that are not very reproducible.
8. Which scientist is more like the typical scientist: AA who produces boring results that are very reproducible or BB who produces exciting results that are not very reproducible.
9. Which scientist is more likely to keep a job: AA who produces boring results that are very reproducible or BB who produces exciting results that are not very reproducible.
10. Which scientist is more likely to get a job: AA who produces boring results that are very reproducible or BB who produces exciting results that are not very reproducible.

**Procedure**

Participants received a link to the study via email. They read a brief description of the study and, if they agreed to participate, completed three tasks in a randomized order. The reputation measures for this study were one of the three tasks. One of the other tasks had individuals rate their ability at verbal tasks before and after completing a set of difficult anagrams. The third task asked participants to judge the morality of an individual who was late to a meeting for different reasons. Those other two tasks are independent studies that were not analyzed in the context of this research. Participants always completed the Responses to Replication portion before completing the Attributes of Researchers portion. At the end of the study, participants answered a few demographic measures, and then were debriefed.

**Results**

**Responses to Replication**

The responses to the initial scenario, that Researcher X found an effect and published it, provide a baseline for comparison with the other scenarios. In the main text, Figure 2 shows the data as Cohen’s *d* effect sizes comparing the baseline with each scenario. In Table A below, we present the means and standard deviations of all conditions.

At baseline, participants provided relatively neutral opinions of both Researcher X and the effect in question, rating her/him as not differently able or ethical compared to the average scientists, and believing the effect to be just as likely to be true as untrue. Compared to baseline, Researcher X was seen as more able and ethical when her/his effect replicated, and the effect itself was judged to be more likely to be true. Conversely, when the effect fails to replicate, Research X is seen as less able and ethical when compared to baseline, and the effect is judged to be slightly less likely to be true.

Beyond simply the result of the replication, Researcher X was judged differently depending on her/his reaction to the replication. For instance, Researcher X was judged as more able and ethical, compared to baseline, when she/he agreed with the failed replication or followed up a failed replication with a new study. In both the former instance, the effect was judged as less likely to be true, and in the latter instance the effect was judged as more likely to be true. Finally, when Research X criticized the failed replication, she/he was judged as less able and less ethical compared to baseline. The effect was also judged to be less likely to be true.

Finally, ratings of Research X were influenced by her/his own actions in following up on the effect. When Researcher X published her/his own failed replication, she/he was judged to be more able and ethical. However, dismissing her/his own failed replication or not following up on the finding greatly reduced ratings of ability and ethicality. In all three of these cases, the effect was seen as less likely to be true.

These results demonstrate that perceptions of researchers are influenced by her/his actions, not solely by the results of the research. Results of studies and replications influenced the perceived likelihood of an effect, but these changes were independent of ratings of researchers. In fact, there were many instances where the researcher was seen as more able and ethical while at the same time her/his effect was seen as less likely to be true. These results support the notion that researcher reputations are more about the process of research rather than the results.

*Table A. Responses to replication, Study 1*

|  | Ability (1-13) | | Ethics (1-13) | | Effect true? (1-11) | |
| --- | --- | --- | --- | --- | --- | --- |
| *Scenario* | M | SD | M | SD | M | SD |
| X found effect and published | 6.98 | 2.21 | 6.78 | 2.21 | 5.98 | 1.8 |
| Y succeeded in replicating | 7.47 | 2.12 | 7.29 | 2.13 | 7.08 | 1.87 |
| Y failed to replicate | 6.6 | 2.27 | 6.62 | 2.26 | 5.62 | 2.05 |
| Y's rep failed, X criticized | 6.03 | 2.45 | 5.82 | 2.52 | 5.48 | 2.02 |
| Y's rep failed, X agreed | 7.47 | 2.24 | 8.12 | 2.48 | 5.35 | 2.12 |
| Y's rep failed, X did new study | 8.15 | 2.27 | 8.27 | 2.3 | 6.19 | 1.72 |
| X published own failed rep | 7.69 | 2.54 | 8.08 | 2.68 | 5.55 | 1.96 |
| X dismissed own failed rep | 5.94 | 2.8 | 5.84 | 3.02 | 5.04 | 2.22 |
| X did not follow up | 5.59 | 2.34 | 5.69 | 2.34 | 5.45 | 1.88 |

**Boring, very reproducible versus Exciting, not very reproducible**

In assigning attributes to scientists, participants favored the scientist that produced boring but reproducible results (Scientist AA) compared to the scientist that produced exciting but not reproducible results (Scientist BB). Scientist AA was more frequently judged to be smarter, more ethical, and a better scientist. In addition, Scientist AA was seen as more like the typical scientist, more likely to get a job, and more likely to keep a job. Participants also more often claimed that they would rather be and should be like Scientist AA. The only dimension that Scientist BB was favored on was creativity, and AA and BB were similarly likely to be assessed as a more celebrated scientist.

*Table B. Attributions of researchers, Study 1. Number of participants selecting AA, BB, or neither.*

| *Attribute* | AA (boring but reproducible) | Neither AA nor BB | BB (exciting but not reproducible) |
| --- | --- | --- | --- |
| Who is smarter? | 631 | 474 | 119 |
| More ethical? | 674 | 470 | 82 |
| A better scientist? | 722 | 399 | 98 |
| More creative? | 364 | 354 | 502 |
| Who would you rather be? | 749 | 211 | 259 |
| Who should you be? | 795 | 257 | 165 |
| More like the typical scientist? | 793 | 268 | 157 |
| The more celebrated scientist? | 509 | 242 | 466 |
| More likely to get a job? | 698 | 256 | 263 |
| More likely to keep a job? | 857 | 236 | 124 |

**Discussion**

The results of Study 1 suggest that, when judging researchers themselves, the process rather than the results is most important. Furthermore, when choosing between exciting results and reproducible results, participants favored the scientist that produced the latter. However, this second finding might have been influenced by our choice of wording. Perhaps, phrasing a scientist’s work as not reproducible was so strong that how boring or exciting an effect was ceased to matter.

**Study 2**

**Method**

**Participants**

Undergraduate students (*N* = 428) were recruited online through the University of Virginia participant pool. Participants received partial course credit in return for participating. Overall, the sample was 51.5% female, the average age was 18.9 years (*SD* = 1.09), and 60% were White, 6.8% Black, 19.2% Asian, 3.8% Hispanic, and 10.2% Other. This sample is much more homogeneous than the Study 1 sample.

**Materials**

The Responses to Replication portion was identical to Study 1. Given the overwhelming preference for the ‘boring but reproducible’ researcher in the Attributions of Researchers section of Study 1, we decided to slightly alter the descriptions of the two researchers. We were concerned that participants were too strongly influenced by attributions of reproducibility. To remove this possibility, in Study 2, Researcher AA was described as producing “boring but certain results” and Researcher BB was described as producing “exciting but uncertain results.” Participants were told: “Imagine two scientists AA and BB who demonstrate different characteristics in the results that they produce from their research. In this context, results that are certain are ones that have been shown to be reliable and reproducible. Results that are uncertain are ones where the reliability and reproducibility are unknown. Reproducible means that the results recur when the study is conducted again.” Participants made the same attributional decisions as in Study 1. Then, in the researcher descriptions for each of the 10 attributes AA and BB were described as: “AA who produces boring but certain results or BB who produces exciting but uncertain results.”

**Procedure**

Participants selected the study from a list of available studies on the University of Virginia’s participant pool website. All studies on this site were given non-descriptive names. The reputation measures were always the first task in the study. The Responses to Replication and Attributions of Researchers portions appeared in a random order. At the end of the study, participants responded to a few demographic measures, and then were debriefed.

**Results**

**Responses to Replication**

Results were similar to Study 1 (see Table C). The veracity of the effect was most influenced by the results of replications, being rated as more likely to be true when the replication succeeded and more likely to be untrue when the replication failed. In addition, Researcher X was judged to be more able and ethical when she/he reacted positively to replications. Participants judged Researcher X as less able and ethical when she/he responded negatively or did not follow up on the original finding. Again, these results support the notion that although effects are judged by results, researchers are judged by process.

*Table C. Responses to replication, Study 2*

|  | Ability (1-13) | | Ethics (1-13) | | Effect true? (1-11) | |
| --- | --- | --- | --- | --- | --- | --- |
| *Scenario* | M | SD | M | SD | M | SD |
| X found effect and published | 7.46 | 1.79 | 6.89 | 1.33 | 6.08 | 1.45 |
| Y succeeded in replicating | 7.95 | 1.79 | 7.36 | 1.69 | 7.43 | 1.75 |
| Y failed to replicate | 6.46 | 1.65 | 6.44 | 1.44 | 4.79 | 1.44 |
| Y's rep failed, X criticized | 6.21 | 1.83 | 5.92 | 1.70 | 5.38 | 1.55 |
| Y's rep failed, X agreed | 7.20 | 1.86 | 8.20 | 2.19 | 4.07 | 1.66 |
| Y's rep failed, X did new study | 8.51 | 1.98 | 8.23 | 1.94 | 5.60 | 1.54 |
| X published own failed rep | 8.05 | 2.13 | 8.66 | 2.29 | 4.74 | 1.67 |
| X dismissed own failed rep | 5.22 | 2.15 | 5.17 | 2.70 | 4.11 | 1.74 |
| X did not follow up | 5.39 | 1.86 | 5.76 | 1.80 | 5.15 | 1.49 |

**Boring, certain versus Exciting, not certain**

As in Study 1, Scientist AA was generally favored over Scientist BB, but less consistently. Participants judged Scientist AA to be smarter, more ethical, a better scientist, more like the typical scientist, more likely to get a job, and more likely to keep a job. Scientist BB was once again favored for creativity, and - in this sample - as being the more celebrated scientist. Finally, participants once again claimed that they should be like Scientist AA, but also claimed that they would rather be like Scientist BB.

*Table D. Attributions of researchers, Study 2*

| *Attribution* | AA (boring but certain) | Neither AA nor BB | BB (exciting but not certain) |
| --- | --- | --- | --- |
| Who is smarter? | 220 | 121 | 41 |
| More ethical? | 221 | 138 | 22 |
| A better scientist? | 199 | 129 | 53 |
| More creative? | 20 | 59 | 302 |
| Who would you rather be? | 145 | 37 | 199 |
| Who should you be? | 214 | 80 | 87 |
| More like the typical scientist? | 301 | 39 | 41 |
| The more celebrated scientist? | 88 | 32 | 261 |
| More likely to get a job? | 212 | 48 | 121 |
| More likely to keep a job? | 309 | 37 | 35 |

**Discussion**

The results of Study 2 were mostly similar to Study 1. Participants judged researchers that responded positively to replications as more able and ethical than those who responded negatively. Furthermore, although less consistently than in Study 1, a scientist that produced boring but certain results was favored more often over a scientist that produced exciting but less certain findings. The differences in attributions between Studies 1 and 2 could have been due to our changed descriptions of the scientists or because of the characteristics of our different sample. To better explore these possibilities, we sought to replicate the results of Study 2 using the same heterogeneous participant source as Study 1 with a very large sample to obtain reliable estimates.

**Study 3**

**Method**

**Participants**

U.S. Participants (*N* = 3,465) were recruited online through a sampling agency (the same as Study 1) and paid in return for completing a 20 minute study. Overall, the sample was 69.2% female, the average age was 35.2 years (*SD* = 14.79), and 82.1% were White, 6.2% Black, 4.6% Asian, 4% Hispanic, and 3.1% Other.

**Materials**

We used the same materials as in Study 2.

**Procedure**

All participants first completed an unrelated study about beliefs in their verbal ability before completing the reputation measures. Participants then completed the Reactions to Replication and Attributions of Researchers portions with the order of the two surveys determined randomly for each participant. Finally, participants provided some demographic information.

**Results**

**Responses to Replication**

Participants provided very similar ratings of Researcher X at baseline compared to Study 1, rating her/him similarly on ability and ethicality to the average scientist, and rating the effect as being just as likely to be true or untrue. The pattern of results, compared to this baseline, mirrors that of Study 1 (see Table E). Researcher X was judged more favorably when she/he responded positively to replications. Conversely, Researcher X was judged less favorable when she/he responded negatively. These ratings were independent of judgments of the effect, which were influenced by whether or not the effect replicated. Again, this supports the notion that researchers are judged more by their research process rather than by their results.

*Table E. Responses to replication, Study 3*

|  | Ability (1-13) | | Ethics (1-13) | | Effect true? (1-11) | |
| --- | --- | --- | --- | --- | --- | --- |
| *Scenario* | M | SD | M | SD | M | SD |
| X found effect and published | 6.96 | 1.99 | 6.73 | 2.01 | 5.94 | 1.63 |
| Y succeeded in replicating | 7.48 | 2.02 | 7.30 | 1.97 | 7.05 | 1.82 |
| Y failed to replicate | 6.50 | 2.07 | 6.58 | 2.01 | 5.50 | 1.75 |
| Y's rep failed, X criticized | 6.20 | 2.16 | 6.00 | 2.28 | 5.55 | 1.78 |
| Y's rep failed, X agreed | 7.24 | 2.16 | 7.99 | 2.37 | 5.12 | 1.96 |
| Y's rep failed, X did new study | 8.03 | 2.15 | 8.18 | 2.22 | 6.07 | 1.56 |
| X published own failed rep | 7.64 | 2.33 | 8.02 | 2.54 | 5.41 | 1.85 |
| X dismissed own failed rep | 6.01 | 2.58 | 5.88 | 2.86 | 5.01 | 2.03 |
| X did not follow up | 5.74 | 2.15 | 5.76 | 2.22 | 5.47 | 1.72 |

**Boring, certain versus Exciting, not certain**

The pattern of attribution results mirrored those of Study 1 (see Table F). Scientist BB was more often judged to be like the more celebrated scientist, though only slightly. Scientist BB was again more often judged to be more creative. Scientist AA was favored on all other attributions.

*Table F. Attributions of researchers, Study 3*

| *Attribution* | AA (boring but certain) | Neither AA nor BB | BB (exciting but not certain) |
| --- | --- | --- | --- |
| Who is smarter? | 1468 | 1289 | 301 |
| More ethical? | 1552 | 1233 | 290 |
| A better scientist? | 1579 | 1261 | 227 |
| More creative? | 734 | 1066 | 1262 |
| Who would you rather be? | 1989 | 549 | 522 |
| Who should you be? | 2053 | 620 | 393 |
| More like the typical scientist? | 1952 | 647 | 466 |
| The more celebrated scientist? | 1169 | 669 | 1218 |
| More likely to get a job? | 1629 | 790 | 637 |
| More likely to keep a job? | 1979 | 687 | 395 |

**Discussion**

The results of Study 3 were similar to the prior studies. This provides consistency to the findings regarding which information is most important for assessing the reputations of researchers and scientists. Of note, the results of the Attributions of Researchers section was largely unaffected by the changing of wording from ‘reproducible’ to ‘certain.’ This suggests that the fluctuations in patterns between Studies 1 and 2 on this section might have been due to sample characteristics rather than wording, or random error as the Study 2 sample was comparatively small.

The first three samples provided consistent opinions on how researchers should be judged. In each, respondents provided an idealized opinion on science, judging researchers by the ways that they conduct research rather than by the results of their research. As such, they could be credited with an overly optimistic view of science. We next investigated whether or not this view matched the opinions of those actually conducting research.

**Study 4**

**Method**

**Participants**

We advertised our study on psychology listservs and through social media. In total, 313 researchers provided at least one response. Overall, the sample was 35.9% female and the average age was 35.4 years (*SD* = 10.44). Most researchers were psychologists (92.3%). For their current position, 15.3% were professors, 14.5% were associate professors, 20.6% were assistant professors, 14.1% were post-docs, 29% were PhD students, and 6.5% were Other.

**Materials**

We used the same materials as in Studies 2 and 3.

**Procedure**

Participants completed the Reactions to Replication and Attributions of Researchers portions in a random order. Participants then provided some demographic information.

**Results**

**Responses to Replication**

Researchers made similar judgments as the general population adult samples from the prior studies. Ratings of researcher reputation (ability and ethics) were distinct from ratings of the truth of the effect. Researcher reputation reflected how Researcher X responded to replication attempts and conducted her/his research regardless of whether a replication succeeded or failed. Conversely, the outcome of replication studies did relate to ratings of how likely it was that the effect was true. Although the pattern of responses resembled the patterns of general adult samples from Studies 1 and 3, researchers tended to make more extreme judgments of Researcher X (larger differences in ratings compared to the baseline scenario), both positively and negatively. For the largest difference in judgments between researchers and the general adult sample, researchers judged Researcher X fairly neutrally (relative to baseline) when she/he did not follow up on an initial finding, whereas adults in the general samples judged Researcher X quite negatively in the same scenario.

*Table G. Responses to replication, Study 4*

|  | Ability (1-13) | | Ethics (1-13) | | Effect true? (1-11) | |
| --- | --- | --- | --- | --- | --- | --- |
| *Scenario* | M | SD | M | SD | M | SD |
| X found effect and published | 7.39 | 1.10 | 6.94 | .66 | 6.44 | 1.34 |
| Y succeeded in replicating | 8.05 | 1.55 | 7.45 | 1.17 | 7.88 | 1.09 |
| Y failed to replicate | 6.95 | 1.08 | 6.86 | .86 | 5.20 | 1.30 |
| Y's rep failed, X criticized | 6.57 | 1.33 | 6.29 | 1.41 | 5.33 | 1.33 |
| Y's rep failed, X agreed | 8.78 | 1.80 | 9.30 | 1.78 | 4.43 | 1.31 |
| Y's rep failed, X did new study | 9.23 | 1.55 | 8.69 | 1.62 | 5.79 | 1.08 |
| X published own failed rep | 9.42 | 1.92 | 10.02 | 1.88 | 4.77 | 1.40 |
| X dismissed own failed rep | 6.33 | 1.46 | 5.86 | 1.85 | 4.80 | 1.36 |
| X did not follow up | 6.95 | .93 | 6.91 | .63 | 6.23 | 1.16 |

**Boring, certain versus Exciting, not certain**

Researchers were more divided in their attributions relative to the general adult samples. *Neither AA (boring but certain) nor BB* *(exciting but not certain)* was the most common answer for who is smarter, more ethical, and the better scientist. Nonetheless, researchers more frequently selected the boring, certain AA over the exciting, uncertain BB, for each of these attributions. In fact, extremely few researchers selected the exciting, uncertain scientist as being more ethical or the better scientist. On the other hand, there was a strong tendency to rate that the exciting, uncertain scientist was more creative. Researchers were more likely to say that they would rather be and should be like the boring, certain scientist, but with a close to even split on rather being the exciting, certain scientist. Also, like the adult samples, scientists assessed the boring, certain scientist to be more typical, and were much more likely to say that the exciting, uncertain scientist was more likely to get a job and be more celebrated, but that tendency was somewhat weaker on whether the exciting, uncertain scientist would be more likely to keep a job.

*Table H. Attributions of researchers, Study 4*

| *Attribution* | AA (boring but certain) | Neither AA nor BB | BB (exciting but not certain) |
| --- | --- | --- | --- |
| Who is smarter? | 62 | 163 | 38 |
| More ethical? | 121 | 151 | 1 |
| A better scientist? | 108 | 149 | 16 |
| More creative? | 13 | 73 | 186 |
| Who would you rather be? | 103 | 73 | 93 |
| Who should you be? | 113 | 108 | 46 |
| More like the typical scientist? | 123 | 61 | 86 |
| The more celebrated scientist? | 13 | 28 | 231 |
| More likely to get a job? | 16 | 26 | 229 |
| More likely to keep a job? | 67 | 72 | 131 |

**Discussion**

Overall, researchers made similar judgments of other scientists as adults and undergraduate students, judging scientists on how they conducted research rather than the results of replications. Furthermore, researchers favored those who discovered boring but certain findings over those who discovered exciting but less certain findings, although this sample was much more divided on these attributions relative to the previous samples.

There were, however, a few differences between researcher responses and responses from the other three samples. Researchers believed that scientists who produced exciting, uncertain results compared to more boring, certain work would have more success in attaining and keeping a job. Also, a scientist who does not follow up on her/his initial findings and moves on to other projects was judged less negatively by researchers compared to other samples. These differences might be the result of experience with research and the research community where replications are not expected or rewarded, especially relative to novel, exciting research. Nonetheless, researchers favored those who produced certain findings and who responded positively to replications when judging qualities related to a researcher’s character and reputation.
